# A Case of Ruptured Hepatocellular Carcinoma As the Initial Presentation of Undiagnosed Hepatitis B-related Cirrhosis in a Young Adult

**DOI:** 10.7759/cureus.102872

**Published:** 2026-02-03

**Authors:** Bassem Al Hariri, Abdelrahman Mostafa, Joudi Alhariri

**Affiliations:** 1 Department of Internal Medicine, Hamad Medical Corporation, Doha, QAT; 2 College of Medicine, Weill Cornell Medicine - Qatar, Doha, QAT; 3 College of Medicine, Qatar University, Doha, QAT; 4 Department of Internal Medicine, Hazm Mebaireek General Hospital, Doha, QAT; 5 College of Medicine, Syrian Private University, Damascus, SYR

**Keywords:** case report, hepatitis b, hepatocellular carcinoma, liver cirrhosis, tumor rupture

## Abstract

Spontaneous rupture of hepatocellular carcinoma (HCC) is a life-threatening oncologic emergency and can be the first manifestation of untreated chronic liver disease. We report the case of a 42-year-old male with no known medical history who presented with severe right upper quadrant and referred shoulder pain. Examination revealed ascites and cachexia. Laboratory tests showed bicytopenia, impaired liver synthetic function, and chronic hepatitis B virus (HBV) infection, despite a normal alpha-fetoprotein (AFP) level. Imaging demonstrated a cirrhotic liver with a large exophytic LI-RADS 5 mass in segment VIII and clear extracapsular rupture. After a multidisciplinary diagnosis of ruptured AFP-negative HCC on a background of undiagnosed Child-Pugh B (score 6) cirrhosis, the patient was stabilized. Antiviral therapy (entecavir) was initiated, and transarterial chemoembolization (TACE) was planned. At discharge, the patient was hemodynamically stable and scheduled for outpatient TACE. This case highlights that ruptured HCC can be a sentinel event in occult cirrhosis, even in younger patients. It underscores the importance of considering HCC in acute abdomen, the limitations of AFP as a sole screening marker (with up to 30% of HCCs being AFP-negative), and the value of a multidisciplinary approach.

## Introduction

Hepatocellular carcinoma (HCC) is the most common primary liver malignancy and a leading cause of cancer-related mortality worldwide [1]. Most HCC cases arise in the setting of cirrhosis due to chronic hepatitis B (HBV), hepatitis C (HCV), alcohol consumption, or non-alcoholic fatty liver disease [2]. Although surveillance programs have improved early detection in high-risk groups, some patients still present with advanced complications. Notably, approximately 30% of HCCs are alpha-fetoprotein (AFP)-negative, which can pose a diagnostic challenge [3]. Spontaneous rupture of HCC is a catastrophic oncologic emergency with an incidence of 3-15% and a high in-hospital mortality rate [4]. It typically manifests with acute abdominal pain and hemoperitoneum and is more common in large, peripherally located tumors at advanced stages [5]. Rupture as the initial presentation of previously undiagnosed liver disease is exceedingly rare and poses a significant diagnostic challenge. This case report aims to highlight this diagnostic pitfall, emphasize the limitations of AFP, and illustrate the critical role of a multidisciplinary team in managing this complex presentation of occult HBV-related cirrhosis.

## Case presentation

A 42-year-old male construction worker with no known past medical history and no prior HBV screening or vaccination presented on November 20, 2025, with a one-day history of acute-onset, severe colicky pain. He reported no history of alcohol use, chronic medications, or known comorbidities, but was an active smoker with a 20-pack-year history and a remote history of left-sided chest tube placement 20 years earlier.

Initial laboratory investigations revealed anemia (hemoglobin 11.2 g/dL), thrombocytopenia (platelets 70,000/µL), impaired synthetic function (albumin 33 g/L, INR 1.4, total bilirubin 22 µmol/L), a normal alpha-fetoprotein level (2 IU/mL), and hepatitis B serology positive for hepatitis B surface antigen (HBsAg), anti-HBc, and anti-HBe, consistent with chronic infection (Table 1). These findings corresponded to a Child-Pugh score of 6 (Class B) and a Model for End-Stage Liver Disease (MELD)-Na score of 13.

**Table 1:** Trend of key laboratory parameters during hospitalization Reference ranges are indicated in brackets. HBV DNA results remained pending throughout the documented hospitalization period, consistent with the lab's turnaround time. The persistent cytopenias and impaired synthetic function underscore the severity of decompensated cirrhosis. INR: international normalized ratio; ALT: alanine transaminase; AST: aspartate aminotransferase; HBsAg: hepatitis B surface antigen

Date	Hb (g/dL) (13.5-17.5)	Platelets (x10³/µL) (150-400)	INR (0.9-1.2)	Albumin (g/L) (35-50)	Total Bilirubin (µmol/L) (3-20)	ALT (U/L) (7-55)	AST (U/L) (8-48)	HBsAg	Anti-HBc	Anti-HBe	HBV DNA (IU/mL)
20-Nov-25	11.2	70	1.4	33	22	56	63	Positive	Positive	Positive	Pending
23-Nov-25	8.0	55	1.4	29	31	48	57	–	–	–	Pending
27-Nov-25	10.3	73	1.4	28	24	56	75	–	–	–	Pending

**Figure 1:**
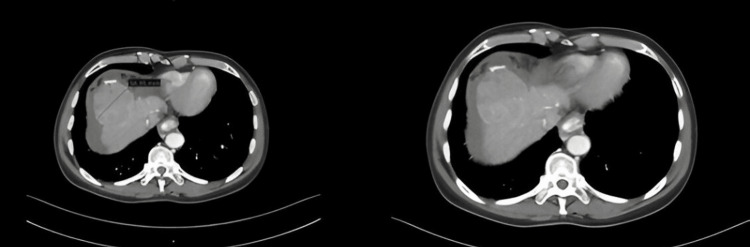
CT abdomen with contrast Showing a large liver lesion in a background of liver cirrhosis, concerning for malignant liver tumors

Contrast-enhanced computed tomography of the abdomen demonstrated a cirrhotic liver with a large (6.6 × 5.2 × 5.2 cm) heterogeneously enhancing exophytic mass in segment VIII, findings suggestive of a tumoral pseudoaneurysm, ascites, and varices (Figure 1).

**Figure 2:**
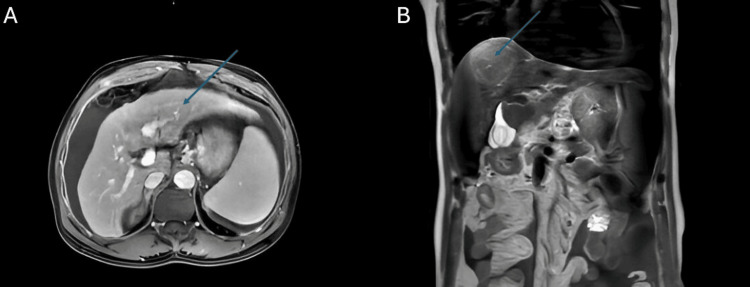
Magnetic resonance imaging of the liver with contrast Panel A: Axial T1-weighted post-contrast image demonstrates a large LI-RADS 5 lesion (arrow) in segment VIII of the right hepatic lobe, showing characteristic arterial phase hyperenhancement and washout, with evidence of extracapsular rupture into the subphrenic space. Panel B: Coronal T2-weighted image reveals the same lesion (arrow), adjacent to ascites. Additional subcentimeter nodules (not shown) elsewhere in the liver were categorized as LI-RADS 3 (probably benign) and LI-RADS 4 (suspicious, not definite for HCC). LI-RADS: Liver Imaging Reporting and Data System

Magnetic resonance imaging of the liver confirmed a Liver Imaging Reporting and Data System (LI-RADS) 5 lesion with arterial hyperenhancement, washout, and delayed capsular enhancement, alongside clear extracapsular rupture into the subphrenic space, additional LI-RADS 3/4 lesions, and peritoneal thickening (Figure 2).

Diagnostic paracentesis yielded hemorrhagic ascitic fluid with a neutrophil count of 380 cells/µL, confirming spontaneous bacterial peritonitis, while esophagogastroduodenoscopy revealed grade II esophageal varices and portal hypertensive gastropathy.

A multidisciplinary team review confirmed the diagnosis of ruptured, alpha-fetoprotein-negative hepatocellular carcinoma on a background of previously undiagnosed hepatitis B virus-related Child-Pugh class B cirrhosis, staged as likely BCLC C (Barcelona Clinic Liver Cancer stage C). Antiviral therapy with entecavir was started indefinitely, and transarterial chemoembolization was planned as the primary locoregional therapy.

## Discussion

This case of a ruptured, AFP-negative HCC revealing occult HBV-related cirrhosis in a young adult provides several critical insights for clinical practice and public health. First, it exemplifies a formidable diagnostic pitfall. The presentation with acute right upper quadrant and referred shoulder pain is a classic mimic of biliary colic or a perforated viscus [5]. In the absence of a known history of liver disease, the subtle signs of chronic illness, cachexia, gum bleeding, and laboratory findings of cytopenias can be easily overshadowed by the acute abdominal emergency. This underscores the imperative for clinicians evaluating acute abdomen, particularly in regions with high HBV prevalence or in patients with demographic risk factors, to maintain a high index of suspicion for ruptured HCC even when overt stigmata of cirrhosis are absent [5].

Second, this case starkly illustrates the silent progression of chronic HBV infection. The patient reached middle age completely unaware of his infectious status, which progressed insidiously to cirrhosis and ultimately to advanced HCC without prior clinical detection. This trajectory reinforces the established yet underutilized public health mandate for universal HBV screening and vaccination [2,3]. Early identification of chronic carriers allows for timely initiation of antiviral therapy, which can significantly slow fibrosis progression, reduce hepatocellular injury, and thereby lower the long-term risk of carcinogenesis [2]. According to contemporary guidelines, indefinite antiviral therapy with potent agents like entecavir or tenofovir is recommended for all patients with HBV-related decompensated cirrhosis, irrespective of viral load, to prevent further hepatic injury and complications [6].

A third, pivotal lesson concerns diagnostic methodology. The patient’s consistently normal AFP level (2 IU/mL) challenges the over-reliance on this biomarker as a standalone screening tool. Contemporary literature confirms that a substantial proportion, up to 30%, of HCCs are AFP-negative [1]. This case validates the primacy of dynamic contrast-enhanced cross-sectional imaging and standardized reporting systems like LI-RADS in diagnosing HCC, irrespective of serologic markers [1].

Finally, the management of ruptured HCC demands a nuanced, multi-pronged strategy that addresses both the immediate emergency and the underlying chronic liver disease. Initial priorities are hemodynamic stabilization and control of active bleeding. For hemodynamically stable patients, as in this case, transarterial chemoembolization (TACE) emerges as a cornerstone intervention. It serves the dual purpose of achieving hemostasis through selective embolization of the tumor's arterial supply while providing regional oncologic control [7,8]. For this patient, TACE was selected over emergency surgical resection given the background of Child-Pugh B cirrhosis and multifocal disease, and over bland arterial embolization due to the added potential oncologic benefit of concurrent localized chemotherapy delivery [7,8].

## Conclusions

This case reinforces three critical clinical lessons: 1) Ruptured HCC can be the initial, dramatic presentation of occult cirrhosis, even in young adults without known liver disease; 2) Diagnosis must rely on robust imaging criteria, as tumor markers like AFP can be normal in a significant minority of cases; 3) Successful management requires a prompt, multidisciplinary strategy that concurrently addresses the oncologic emergency and the underlying decompensated cirrhosis. Heightened clinical suspicion in at-risk patients presenting with an acute abdomen is paramount.
